# The SCO2102 Protein Harbouring a DnaA II Protein-Interaction Domain Is Essential for the SCO2103 Methylenetetrahydrofolate Reductase Positioning at *Streptomyces* Sporulating Hyphae, Enhancing DNA Replication during Sporulation

**DOI:** 10.3390/ijms23094984

**Published:** 2022-04-30

**Authors:** Gemma Fernández-García, Nathaly González-Quiñónez, Beatriz Rioseras, Sergio Alonso-Fernández, Javier Fernández, Felipe Lombó, Ángel Manteca

**Affiliations:** Área de Microbiología, Departamento de Biología Funcional, IUOPA and ISPA, Facultad de Medicina, Universidad de Oviedo, 33006 Oviedo, Spain; gemmafg06@hotmail.com (G.F.-G.); natygq@gmail.com (N.G.-Q.); brioseras@gmail.com (B.R.); sergioalonsofernandez@gmail.com (S.A.-F.); uo186966@uniovi.es (J.F.); lombofelipe@uniovi.es (F.L.)

**Keywords:** *Streptomyces*, DnaA, SCO2102 DnaA homologue, SCO2103 MTHFR, sporulation, differentiation, cell division

## Abstract

*Streptomyces* DNA replication starts with the DnaA binding to the origin of replication. Differently to most bacteria, cytokinesis only occurs during sporulation. Cytokinesis is modulated by the divisome, an orderly succession of proteins initiated by FtsZ. Here, we characterised SCO2102, a protein harbouring a DnaA II protein–protein interaction domain highly conserved in *Streptomyces*. The *ΔSCO2102* knockout shows highly delayed sporulation. SCO2102-mCherry frequently co-localises with FtsZ-eGFP during sporulation and greatly reduces FtsZ-eGFP Z-ladder formation, suggesting a role of SCO2102 in sporulation. *SCO2102* localises up-stream of *SCO2103*, a methylenetetrahydrofolate reductase involved in methionine and dTMP synthesis. *SCO2102/SCO2103* expression is highly regulated, involving two promoters and a conditional transcription terminator. The *ΔSCO2103* knockout shows reduced DNA synthesis and a non-sporulating phenotype. SCO2102-mCherry co-localises with SCO2103-eGFP during sporulation, and SCO2102 is essential for the SCO2103 positioning at sporulating hyphae, since SCO2103-eGFP fluorescent spots are absent in the *ΔSCO2102* knockout. We propose a model in which SCO2102 positions SCO2103 in sporulating hyphae, facilitating nucleotide biosynthesis for chromosomal replication. To the best of our knowledge, SCO2102 is the first protein harbouring a DnaA II domain specifically found during sporulation, whereas SCO2103 is the first methylenetetrahydrofolate reductase found to be essential for *Streptomyces* sporulation.

## 1. Introduction

Streptomycetes are important biotechnological bacteria from which two thirds of the bioactive secondary metabolites used in clinics (mainly antibiotics, but also antitumourals, immunosuppressors, etc.) have been discovered [1,2]. They have complex developmental cycles that include programmed cell death, hyphae differentiation and sporulation [3,4]. One of the most intriguing processes in *Streptomyces* biology is cell division, since this bacterium grows in the form of multinucleated hyphae with sporadic septae that, at the end of the development, form individual spores (reviewed in [5]).

Bacterial chromosomal replication is led by the binding of the DnaA AAA + ATPase to the origin of replication (oriC). DnaA is a complex protein constituting four structural domains [6,7]: domain I participates in orisome formation, domain II modulates protein–protein interaction, domain III participates in ATP binding, and domain IV controls DNA binding [6,7,8]. Chromosomal replication usually continues with the formation of the divisome, formed by the proteins that control cell envelope invagination during cytokinesis (reviewed in [9]). The bacterial divisome is a dynamic structure in which several proteins interact in a highly regulated sequence leading to cell division [9]. More than 20 proteins have been described in the model *E. coli* or *Bacillus subtillis* divisomes, yet the precise composition of the bacterial divisome has not been fully characterised [9]. *Streptomyces* DNA replication is unique since the chromosome is not segregated into two daughter cells until sporulation. Consequently, the *Streptomyces* oriC and DnaA proteins show important peculiarities, such as the presence of two DNA-unwinding elements (DUE) [10] or being modulated by Ser-Thr-Tyr phosphorylation [8], respectively.

The tubulin-like FtsZ cell division marks the point at which the divisome is formed [9]. *Streptomyces* has two types of divisomes, one controlling vegetative growth at the substrate and aerial mycelium stages, decoupled from cytokinesis and leading to the formation of multinucleated hyphae, and another one controlling sporulation [5]. *Streptomyces* vegetative and sporulation divisomes share some proteins, such as tubulin-like GTPase FtsZ, which polymerises to form a dynamic ring-like structure known as the Z-ring [11]. However, they differ in other proteins, such as FtsI, FtsL, FtsW, SsgA, SsgB and CrgA, which are required for sporulation but not for vegetative septa formation [5,12,13,14]. Mutation in several *Streptomyces* core divisome genes (*divIC*, *ftsL*, *ftsI*, *ftsQ*, and *ftsW*) results in viable mutant strains with partially functional sporulation, suggesting that other unknown proteins might perform redundant functions [15].

Here, we further characterise DNA replication and cell division during sporulation. We discovered *SCO2102*, a protein harbouring a DnaA II protein-interaction domain highly conserved in the *Streptomyces* genus and highly expressed during sporulation, which co-localises with FtsZ in sporulating hyphae. We also identified that SCO2103*,* previously reported in *S. lividans* as a methylenetetrahydrofolate reductase (MTHFR) involved in methionine [16] and dTMP biosynthesis [17], co-localises with SCO2102 during sporulation, enhancing chromosomal DNA synthesis at sporulating hyphae.

## 2. Results

### 2.1. SCO2102/2103 Genes Are Highly Conserved in Streptomyces and Expressed during Sporulation

**Figure 1:**
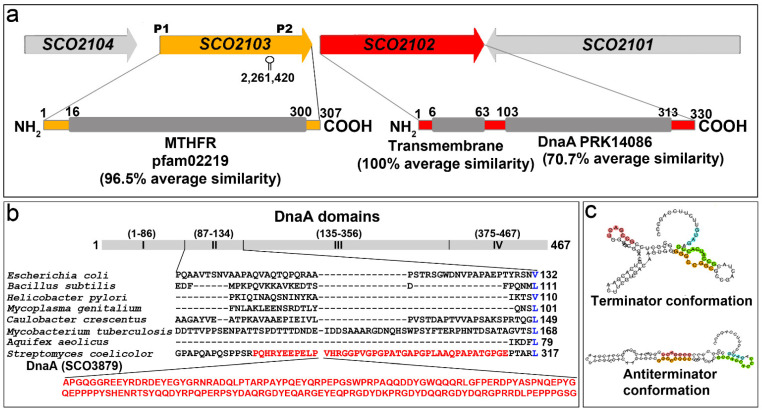
*SCO2102/2103* genetic region, gene homologies and regulatory elements. (**a**) *SCO2102/03* chromosomal region and homologies. (**b**) Alignment of DnaA proteins from different model bacteria (adapted from Zawilak-Pawlik et al. [7]). Amino acid numbering in the scheme correspond to *E. coli* DnaA [6]. The *S. coelicolor* DnaA (SCO3879) region homologue to the *SCO2102* DnaA domain is highlighted in red. (**c**) Terminator/antiterminator conformations of the conditional terminator identified in the *SCO2103* ORF (predicted using the online algorithm developed by Millman et al. [18]).

SCO2102 and SCO2103 show an average amino acid similarity of 72.5 and 96.14%, respectively, among the *S. griseus*, *S. avermitillis*, *S. lividans*, *S. clavuligerus* and *S. venezuelae* model strains. SCO2102 harbours a putative transmembrane domain (100% average similarity in *Streptomyces*) at its amino end, and a DnaA domain (70.7% average similarity in *Streptomyces*) centred in the protein (Figure 1a). As introduced above, the canonical *S. coelicolor* DnaA chromosomal replication-initiator protein (SCO3879) harbours four domains [6]. The SCO2102 *dnaA* domain shows a 46.6% similarity with the SCO3879 *dnaA* II domain involved in protein–protein interaction [7]. Interestingly, the *S. coelicolor* DnaA II domain presents an insertion of 160 amino acids not present in other bacteria [7] (Figure 1b); this additional region is also present in the SCO2102 protein (the SCO3879 region homologous to SCO2102 is highlighted in red in Figure 1b; the SCO2102 and SCO3879 amino acid sequence alignment is shown in Figure S1). 

The SCO2103 protein harbours a conserved MTHFR domain (96.5% average similarity in *Streptomyces*) (Figure 1a). Interestingly, *SCO2102/2103* synteny was maintained in all the above *Streptomyces* model strains. The *SCO2102/2103* gene transcription is complex and modulated by at least two promoters. The *S. lividans SCO2103* orthologue is transcribed from a leaderless mRNA (labelled as P1 in Figure 1a) [16]. Jeong et al. [19] described the existence of at least one other promoter located in the *S. coelicolor SCO2103* ORF (P2 in Figure 1a). *SCO2102/2103* transcription might be even more complex; our bioinformatic analysis using the online algorithm developed by Millman et al. [18] revealed the existence of two mutually exclusive putative terminator and antiterminator RNA structures just before P2 (Figure 1c). 

**Figure 2:**
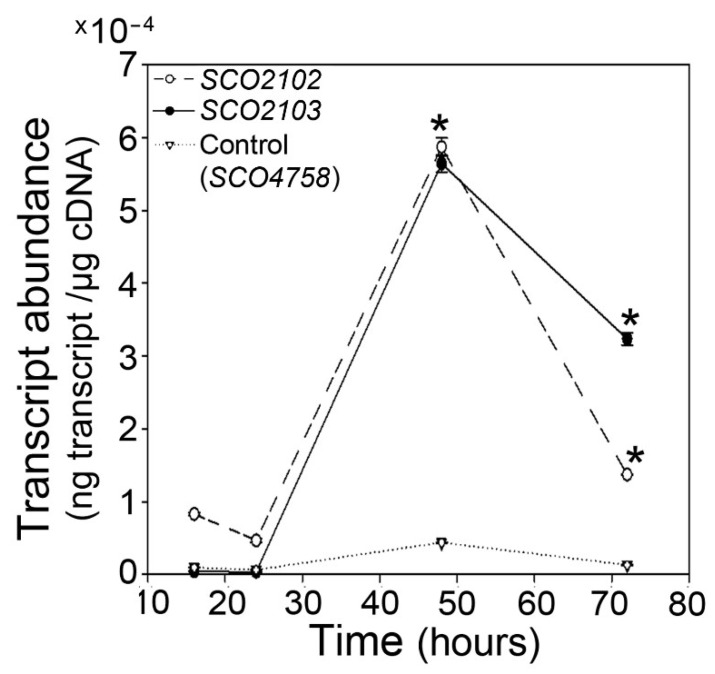
*SCO2102/2103* gene expression in the *S. coelicolor* wild-type strain. *SCO2102* and *SCO2103* transcript abundance in GYM solid cultures. Asterisks indicate significant differences compared to the 16 h sample. Transcript abundance of the *SCO4758* housekeeping gene [20] was used as control. Three biological replicates were processed.

Both genes, *SCO2102* and *SCO2103*, are more highly expressed at the aerial mycelium sporulating hyphae (48 h in GYM) compared to at the substrate mycelium (16 and 24 h in solid GYM cultures) (Figure 2).

### 2.2. ΔSCO2103 Knockout Shows a Bald Phenotype

**Figure 3:**
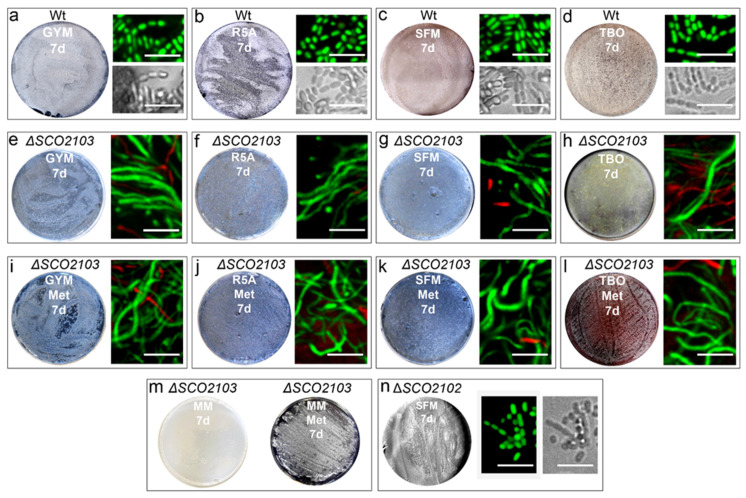
Δ*SCO2102/SCO2103* sporulation on solid cultures. (**a**–**d**) Wild-type in different culture media. (**e**–**h**) Δ*SCO2103* bald phenotype in different culture media. (**i**–**l**) Δ*SCO2103* bald phenotype in methionine-amended media. (**m**) Absence of growth of Δ*SCO2103* in minimal medium without methionine. (**n**) Δ*SCO2102* sporulation growing on SFM solid cultures. Macroscopic view of the Petri plates (grey colour indicates sporulation), laser-scanning confocal fluorescence images of hyphae stained with SYTO9 and PI and phase-contrast mode images are shown. Representative images from at least three biological replicates are shown. Scale bars indicate 5 µm.

The *ΔSCO2103* knockout mutant shows a bald phenotype, i.e., the absence of aerial mycelium (white colour in culture plates) and sporulation (grey colour in culture plates) in all culture media analysed (GYM, R5A, SFM, TBO) (compare the wild-type strain, Figure 3a–d, with the *ΔSCO2103* knockout, Figure 3e–h). Spore chains were never observed in the Δ*SCO2103* hyphae (see laser-scanning confocal fluorescence and phase-contrast images in Figure 3). Actinorhodin production (blue colour in Petri plates) seems to be higher in GYM, R5A, and SFM solid cultures of the Δ*SCO2103* mutant than in the wild-type strain cultures, which might be a consequence of the absence of sporulation: wild-type sporulating hyphae would stop metabolism and antibiotic production, while Δ*SCO2103* non-sporulating hyphae would produce antibiotics for a longer time. Since the *S. lividans SCO2103* orthologue has a methylenetetrahydrofolate reductase involved in methionine synthesis [16], we investigated whether methionine might restore the *S. coelicolor ΔSCO2103* knockout mutant phenotype. However, sporulation was not restored in all methionine-amended media (Figure 3i–l). As expected, the *ΔSCO2103* knockout mutant was unable to grow in minimal medium without methionine (Figure 3m), corroborating the finding that SCO2103 methylenetetrahydrofolate reductase activity participates in methionine biosynthesis [16].

### 2.3. SCO2102 and SCO2103 Gene Expression Is Highly Regulated, Involving at Least Two Promoters and a Conditional Transcriptional Terminator

**Figure 4:**
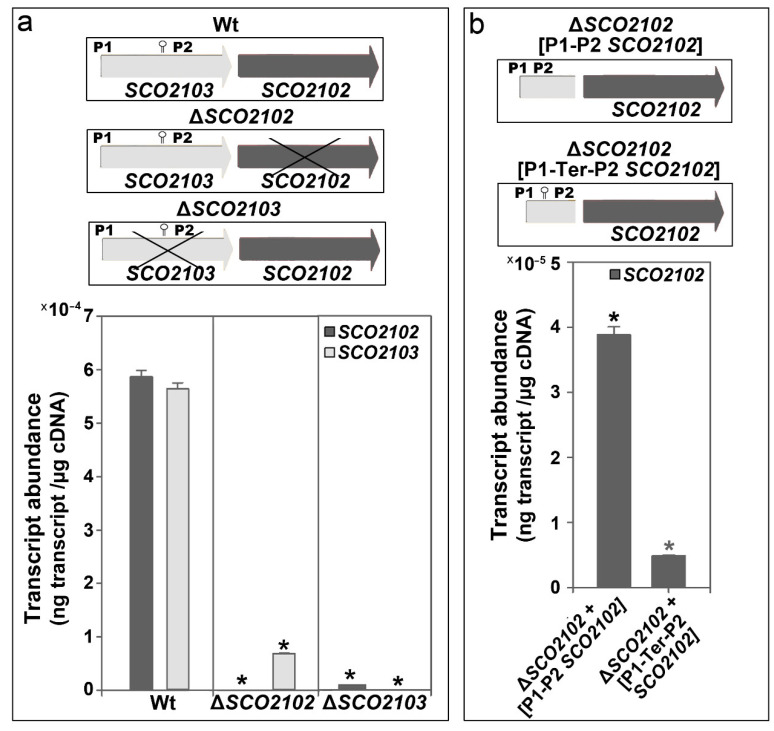
*SCO2102/2103* transcript abundances in solid GYM 48 h cultures. (**a**) *SCO2102/2103* transcript abundances in the wild-type and the Δ*SCO2102/SCO2103* knockout mutants. (**b**) *SCO2102* transcript abundances in the Δ*SCO2102* knockout complemented with the *SCO2102* gene controlled by P1 and P2 with and without the conditional transcriptional terminator. Asterisks indicate significant differences compared to the wild-type strain. Three biological replicates were processed.

In contrast to *ΔSCO2103*, the *ΔSCO2102* knockout mutant can sporulate (Figure 3n). As mentioned above, the expression of the *SCO2102* and *SCO2103* genes is controlled by at least two promoters (P1 and P2 in Figure 1a) [16,19]. Bioinformatics revealed the existence of a putative conditional transcriptional terminator between P1 and P2 (Figure 1c). To further understand the interaction between *SCO2102* and *SCO2103* gene transcription, we quantified their mRNA abundances in the wild-type strain and the Δ*SCO2102* and Δ*SCO2103* knockout mutants growing on solid GYM 48 h cultures (Figure 4a), the time-point showing the maximum *SCO2102* and *SCO2103* transcriptions (Figure 2). We also quantified *SCO2102* mRNA abundance in the Δ*SCO2102* mutant complemented with *SCO2102* and controlled by promoters 1 and 2 (Δ*SCO2102* (pNG3-p1-p2-*SCO2102*) strain) and with *SCO2102* and P1, P2, as well as the putative conditional terminator (Δ*SCO2102* (pNG3-P1-Ter-P2-*SCO2102*)) (Figure 4b).

As expected, the Δ*SCO2102* mutant lacked *SCO2102* expression, whereas the Δ*SCO2103* mutant lacked *SCO2103* transcription (Figure 4a). The Δ*SCO2103* mutant had highly reduced *SCO2102* expression (98% less than the wild-type strain), indicating the importance of P2 in the expression of this gene (Figure 4a). Interestingly, *SCO2103* expression was highly reduced in the Δ*SCO2102* mutant (87% less than the wild-type strain) (Figure 4a), indicating that further unknown regulation, beyond the P1 and P2 promoters, exists.

The identified conditional terminator is functional, as the expression of the *SCO2102* gene in the Δ*SCO2102* mutant complemented with *SCO2102* under the control of the two promoters and the conditional terminator (Δ*SCO2102* (pNG3-P1-Ter-P2-*SCO2102*) strain) was 33% lower than in the construction without the putative conditional terminator (Δ*SCO2102* (pNG3-p1-p2-*SCO2102*) strain) (Figure 4b). Interestingly, *SCO2102* expression in the Δ*SCO2102* mutant complemented with P1 and P2-*SCO2102* was around 10 times lower than that in the wild-type strain (3.8 × 10^-5^ vs. 5.9 × 10^-4^, respectively, Figure 4). This result again indicates the existence of further unknown regulation that could not be reproduced in the Δ*SCO2102* mutant complemented with P1 and P2-*SCO2102*.

### 2.4. ΔSCO2103 Knockout Mutant Sporulation Is Restored by the SCO2103 Gene

**Figure 5:**
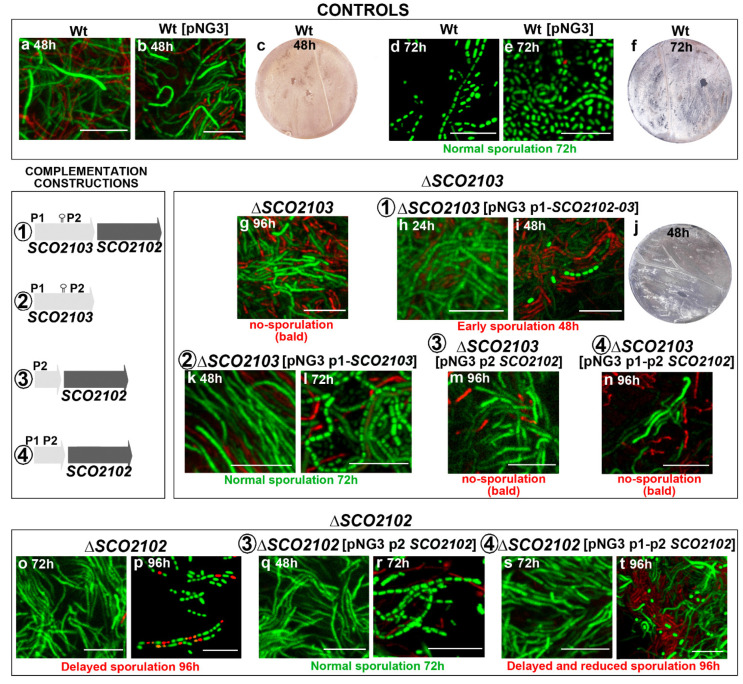
Sporulation timing in GYM solid cultures of the Δ*SCO2102/SCO2103* mutants and their complemented strains. (**a**–**f**) Control cultures of the wild-type strain with and without pNG3. (**g**–**n**) Δ*SCO2103* sporulation with and without different combinations of P1, P2 and the *SCO2102-03* genes cloned into pNG3. (**o**–**t**) Δ*SCO2102* sporulation with and without different combinations of P1, P2 and *SCO2102* cloned into pNG3. Normal sporulation (72 h culture) is labelled in green. The constructions used to complement the Δ*SCO2102/SCO2103* mutants are outlined and numbered as 1–4. Delayed sporulation is labelled in red. Macroscopic view of the Petri plates (grey colour indicates sporulation), and laser-scanning confocal fluorescence images of hyphae stained with SYTO9 and PI are shown. Representative images from at least three biological replicates are shown. Scale bars indicate 8 µm.

As stated above, *SCO2103* ORF removal also affects *SCO2102* due to the elimination of one of the known promoters of *SCO2102* (P2 in Figure 1a), considerably reducing *SCO2102* expression (Figure 4a). Four combinations of the *SCO2102/2103* genes with the P1 and P2 promoters and the putative conditional transcriptional terminator were used in this study to complement the Δ*SCO2102* and Δ*SCO2103* knockout mutants (outlined in Figure 5). The *SCO2103* + (pNG3-p1-SCO2103) strain (Construction 2 in Figure 5) restored wild-type sporulation at 72 h (compare Figure 5d,e with Figure 5l). However, when both *SCO2102* and *SCO2103* genes were used together (Construction 1 in Figure 5), sporulation was accelerated to 48 h (Figure 5i,j). The *SCO2102* gene alone was not able to restore Δ*SCO2103* sporulation (Figure 5m,n). These results indicate that *SCO2103* is the gene responsible for the bald phenotype and that an increase in the dose of the *SCO2102* gene in the Δ*SCO2103* mutant complemented with *SCO2102* and *SCO2103* accelerates sporulation timing.

### 2.5. ΔSCO2102 Knockout Shows a Delay in Sporulation, Which Is Complemented by the SCO2102 Gene

The Δ*SCO2102* knockout mutant was complemented with *SCO2102* under the control of one or two promoters (constructions 3 and 4 in Figure 5). Δ*SCO2102* sporulation was delayed to 96 h in GYM (Figure 5p) compared to the 72 h in the wild-type strain (Figure 5d,e). This delay in sporulation was complemented with *SCO2102* controlled by P2 (Figure 5r). Interestingly, when the two promoters were used in the complementation, the delay in sporulation was not complemented and it was even more delayed than in the Δ*SCO2102* mutant, since after 96 h, culture spores were hardly observable (Figure 5t). This result again indicates an intriguing dose-dependent effect of SCO2102 on sporulation.

### 2.6. Actinorhodin and Undecylprodigiosin Productions Were Altered in the ΔSCO2102 and ΔSCO2103 Knockout Mutants

**Figure 6:**
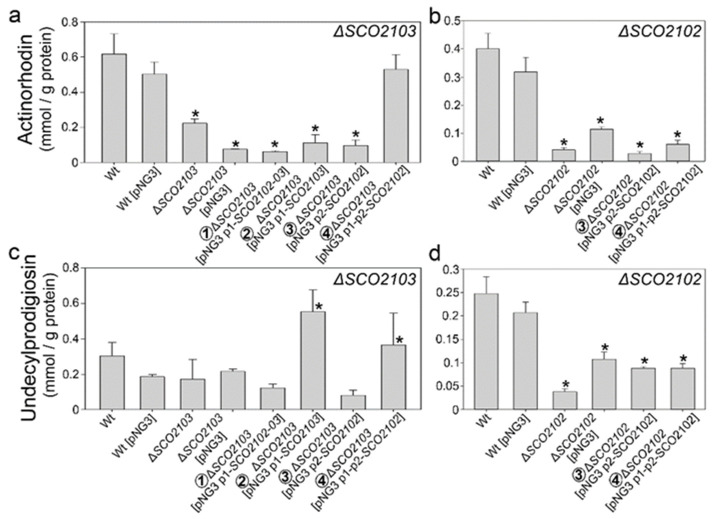
Antibiotic production in Δ*SCO2102,* Δ*SCO2103* and their complemented strains. Antibiotic production was measured in three biological replicates from liquid sucrose-free R5A medium at 168 h once the maximum production was reached. (**a**,**b**) Actinorhodin. (**c**,**d**) Undecylprodigiosin. The constructions used to complement the Δ*SCO2102/SCO2103* mutants are numbered as in Figure 5. Significant differences compared to the wild-type strain are labelled by asterisks.

Actinorhodin production was slightly reduced in the *ΔSCO2103* knockout in liquid sucrose-free R5A non-sporulating cultures (Figure 6a). This phenotype is complemented by the *SCO2102* gene controlled by P1 and P2 (Construction 4 in Figure 5), but not by the other complementation constructions (Figure 6a). Undecylprodigiosin production was not significantly altered in the *ΔSCO2103* knockout compared to the wild-type strain (Figure 6c). Interestingly, some of the *ΔSCO2103*-complemented strains (constructions 2 and 4 in Figure 5) had increased undecylprodigiosin production (Figure 6c).

Actinorhodin and undecylprodigiosin production were considerably reduced in the Δ*SCO2102* mutant (Figure 6b,d). Antibiotic production could not be restored to wild-type levels with the *SCO2102* gene being controlled by one or two promoters (Figure 6b,d).

### 2.7. SCO2102 Co-Localises with FtsZ during Sporulation

**Figure 7:**
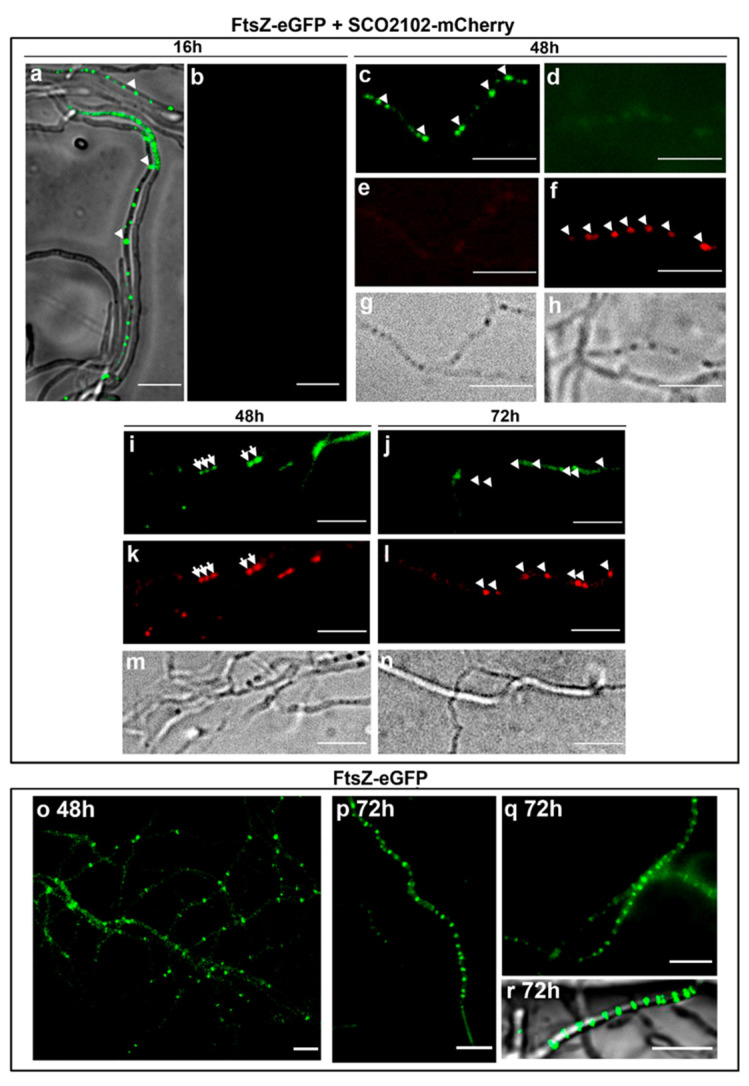
FtsZ-eGFP and SCO2102-mCherry dynamics in sporulating SFM solid cultures. (**a**–**n**) FtsZ-eGFP (green) and SCO2102-mCherry (red) co-expression and cellular localisation in SFM solid cultures. Arrows indicate co-localising FtsZ-eGFP and SCO2102-mCherry spots. Arrow heads indicate FtsZ-eGFP or SCO2102-mCherry spots that do not co-localise. (**o**–**r**) FtsZ-eGFP (green) expression in the *S. coelicolor* wild-type strain. Z-ladders are observed during sporulation (72-h culture). Fluorescence and phase-contrast microscope images are shown. Scale bars indicate 5 µm.

To determine whether SCO2102 co-localises with FtsZ, we created a *SCO2102-mCherry* gene that was introduced into the *S. coelicolor* FM145 strain harbouring *ftsZ-eGFP* created by Willemse and Wezel [21]. We followed SCO2102-mCherry (red) and FtsZ-eGFP (green) during development under a fluorescence microscope (Figure 7). At early time points (16 h), FtsZ-eGFP spots were observed inside some hyphae (arrowheads in Figure 7a), but there was no trace of mCherry fluorescence. At 48 h, when DNA replication associated with sporulation starts in *S. coelicolor* GYM-confluent cultures [22], some hyphae showed eGFP-FtsZ- or SCO2102-mCherry-fluorescent spots (arrowheads in Figure 7c,f). There were also hyphae in which green and red spots co-localised (arrows in Figure 7i,k), indicating that FtsZ and SCO2102 can co-localise during part of the complex succession of proteins modulating sporulation. Under the culture conditions, developmental time points and the fluorescence microscope settings detailed in the methods, there was no detectable autofluorescence in the *S. coelicolor* wild-type control cultures (Figure S2). Although SCO2102-mCherry and eGFP-FtsZ fluorescence was not abundant, it was unequivocally above the fluorescence of the controls observed under the same conditions.

The FtsZ ladders (the succession of 1 µm-separated Z-rings) were easily observed in the *S. coelicolor* FM145 strain expressing FtsZ-eGFP (Figure 7p–r), but we never observed them in sporulating hyphae of the *S. coelicolor* strain expressing FtsZ-eGFP and SCO2102-mCherry (Figure 7j). This result indicates some interference between SCO2102-mCherry and FtsZ-eGFP which precludes, or at least diminishes, FtsZ-eGFP polymerisation.

### 2.8. SCO2102 and SCO2103 Co-Localise during Sporulation

**Figure 8:**
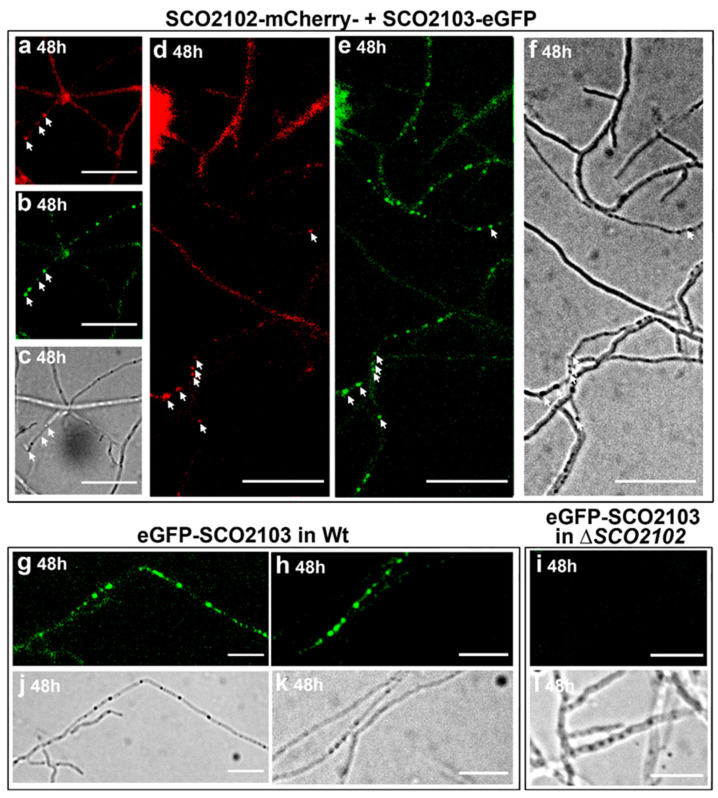
SCO2102-mCherry and SCO2103-eGFP dynamics in sporulating SFM solid cultures. (**a**–**f**) SCO2103-eGFP (green) and SCO2102-mCherry (red) cellular localisation in the *S. coelicolor* wild-type strain. All SCO2102-mCherry spots co-localise with SCO2103-eGFP spots (arrows), but there are SCO2103-eGFP spots that do not co-localise with SCO2102-mCherry spots. (**g**–**l**) SCO2103-eGFP expressed in the wild-type and the Δ*SCO2102* knockout strains. Fluorescence and phase-contrast microscope images are shown. Scale bars 5 µm.

Subsequently, we analysed whether SCO2102 and SCO2103 co-localise, investigating the dynamics of SCO2102-mCherry and SCO2103-eGFP in *S. coelicolor* (Figure 8a–f). Interestingly, all SCO2102-mCherry spots observed at the sporulating hyphae (48 h) co-localised with SCO2103-eGFP (arrows in Figure 8d,e). In contrast, there were several SCO2103-eGFP spots that did not co-localise with SCO2102-mCherry (Figure 8e). The SCO2103-eGFP spots were observed in the wild-type strain (Figure 8g,h), but they were never observed in the Δ*SCO2102* knockout mutant expressing SCO2103-eGFP (Figure 8i). These results reveal that SCO2102 is essential for the SCO2103 positioning at sporulating hyphae. The wild-type control strain observed under the same fluorescence microscope settings did not show observable fluorescence (Figure S2).

### 2.9. Chromosomal DNA Is Reduced in the Hyphae of the ΔSCO2103 Knockout Compared to the ΔSCO2102 Knockout and the Wild-Type Strains

**Figure 9:**
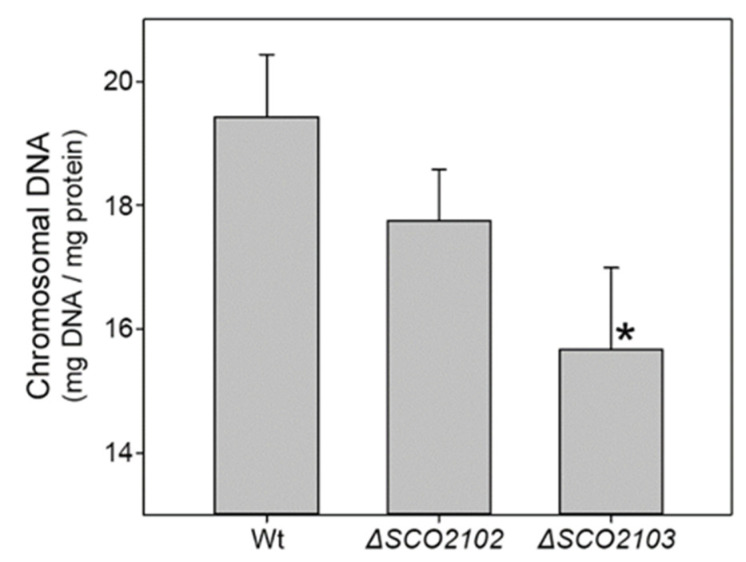
Chromosomal DNA at the wild-type sporulation time points in GYM cultures of the wild-type strain and the Δ*SCO2102/2103* knockout mutants. Significant difference compared to the wild-type strain is labelled by an asterisk.

SCO2103 methylenetetrahydrofolate reductase participates in methionine biosynthesis [16], which was corroborated in this study (Figure 3m). Methylenetetrahydrofolate reductases also participate in other biosynthetic pathways, such as dTTP biosynthesis [17], which is necessary for DNA replication. We tested whether SCO2103 participates in DNA biosynthesis during sporulation, comparing the amounts of chromosomal DNA in the *SCO2102* and *SCO2103* knockout mutants and the wild-type strain (Figure 9). The amount of chromosomal DNA was highly reduced in the Δ*SCO2103* mutant hyphae compared to the wild-type strain (19.4% reduction), indicating a role of SCO2103 in DNA synthesis, necessary for sporulation. Chromosomal DNA was also reduced in the Δ*SCO2102* mutant; however, the difference with the wild-type strain was not significant (Figure 9). A reduction in chromosomal DNA in the Δ*SCO2102* mutant would correlate with the reduction observed in the *SCO2103* expression in this mutant (see above and Figure 4a).

## 3. Discussion

The expression of the *SCO2103*-*SCO2102* genes is modulated by at least two promoters identified by Blanco et al. [16] and Jeong et al. [19] (P1 and P2 in Figure 1a). Here, we identified a conditional terminator upstream of P2 and inside the *SCO2103* ORF (Figure 1c), which reduces *SCO2102* expression when present (Figure 4b). The antiterminator conformation would be necessary for *SCO2103* ORF expression. Conditional transcription termination is a strategy of cis-acting RNA-based regulation consisting of two mutually exclusive terminator and antiterminator RNA structures, that depending on a specific signal (small ligands, depletion of amino acids, antibiotics), can activate premature transcription termination [18]. Further work is necessary to characterise the signal/s modulating the conditional terminator/antiterminator identified in the *SCO2103* ORF. Although *SCO2102* is downstream to *SCO2103*, *SCO2103* expression was highly reduced in the Δ*SCO2102* mutant (87% less compared to the wild-type strain) (Figure 4a), indicating that additional unknown regulation, beyond the P1 and P2 promoters and the conditional terminator, exists. This complex regulation modulates the *SCO2102* and *SCO2103* transcript dose producing a delay (Figure 5s,t) or acceleration (Figure 5i) of the sporulation in our Δ*SCO2102-* and Δ*SCO2103*-complemented strains. Interestingly, we were unable to create the *SCO2102/2103* double mutant, indicating that the combined activity of the SCO2102 and SCO2103 proteins might be essential for *Streptomyces* viability. We were also unable to overexpress *SCO2102*, again suggesting that the *SCO2102* transcript dose might determine development and viability.

In our study, SCO2102 frequently co-localised with FtsZ (SCO2082) during sporulation (Figure 6); FtsZ is the first component of the vegetative and sporulation divisomes, i.e., the proteins that control cell envelope invagination during cytokinesis (reviewed in [9]). We were unable to determine the precise FtsZ and SCO2102 kinetics and we do not know if FtsZ starts first or continues after SCO2102 accumulation in sporulating hyphae. However, our results indicate that SCO2102 participates, together with FtsZ, in the complex succession of proteins regulating *Streptomyces* sporulation [9]. The SCO2102 always co-localised with SCO2103 during sporulation (Figure 8d,e), and positions the SCO2103 methylenetetrahydrofolate reductase at sporulating hyphae, since SCO2103-eGFP fluorescent spots are absent in the *ΔSCO2102* knockout (Figure 8i). Methylenetetrahydrofolate reductases participate in the biosynthesis of dTTP [17], and SCO2103 might be crucial to provide the dTTP necessary for DNA biosynthesis accompanying sporulating hyphae. The amount of chromosomal DNA was, in fact, significantly lower in the Δ*SCO2103* non-sporulating mutant (Figure 9).

Actinorhodin and undecylprodigiosin productions were affected in the Δ*SCO2102/03* knockout mutants (Figure 6). This effect on antibiotic production might be consequence of the reduction in SCO2103 MTHFR-dependant methionine synthesis, since methionine is important for the synthesis of many antibiotics (e.g., via SAM), including acinorhodin [23]. Moreover, methionine visually impacts undecylprodigiosin production (red colour) in the Δ*SCO2103* knockout mutant growing on TBO medium (Figure 3l). The effect on antibiotic production might also be indirect, due to the effect on sporulation timing, since hypha differentiation, sporulation and antibiotic production are highly interconnected [24,25].

**Figure 10:**
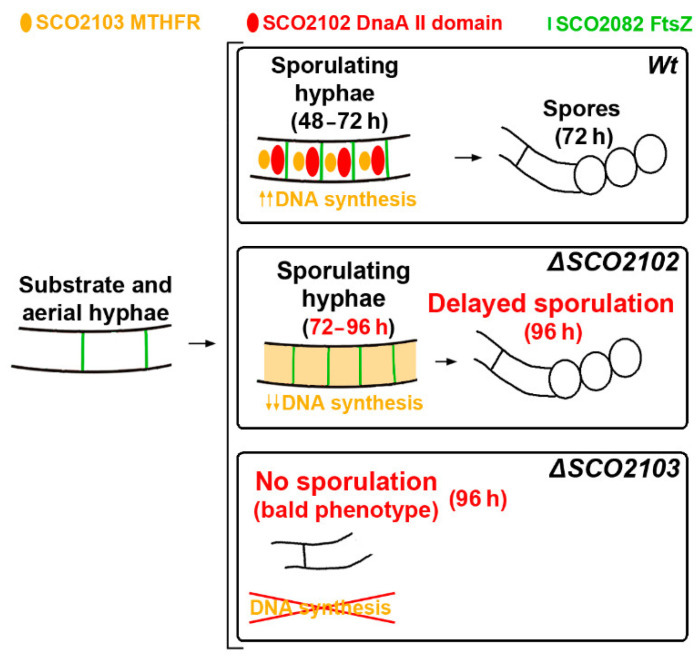
Model outlining the dynamics of FtsZ (green), SCO2102 (red) and SCO2103 (orange) in sporulating hyphae. SCO2102 and FtsZ co-localise in the wild-type sporulating hyphae. SCO2102 is essential for positioning SCO2103 at sporulating hyphae, increasing MTHFR activity and nucleotide synthesis (dTMP), enhancing chromosomal DNA replication accompanying sporulation. SCO2103 is not positioned at sporulating hyphae in the Δ*SCO2102* mutant, reducing dTMP accessibility and delaying sporulation. The absence of SCO2103 MTHFR activity in the Δ*SCO2103* mutant would block the DNA synthesis enhanced by SCO2103, generating a non-sporulating phenotype.

Overall, the main finding of this study is that SCO2102 (harbouring a DnaA II protein-interaction domain) is essential for the positioning of SCO2103 methylenetetrahydro-folate reductase at sporulating hyphae, and that SCO2103 enhances DNA replication during sporulation. Based on these results, we propose a model in which SCO2102 positions SCO2103 at sporulating hyphae, facilitating the nucleotide biosynthesis necessary for chromosomal DNA replication (Figure 10). The absence of SCO2102 in the Δ*SCO2102* mutant precludes SCO2103 localisation at sporulating hyphae, probably limiting dTMP synthesis [17] and delaying sporulation. The Δ*SCO2103* mutant lacks SCO2103 MTHFR activity and displays a strong reduction in DNA synthesis, which would block sporulation. Further work will be necessary to fully understand the interaction between these proteins during sporulation. We can conclude that SCO2102 and SCO2103 cooperate in the modulation of differentiation and sporulation. Normal sporulation requires correct levels of *SCO2102* and *SCO2103* transcription. To the best of our knowledge, SCO2102 is the first protein harbouring a DnaA II domain specifically found during sporulation, whereas SCO2103 is the first methylenetetrahydrofolate reductase found to be essential for *Streptomyces* sporulation.

## 4. Materials and Methods

### 4.1. Bacterial Strains and Culture Conditions

All *Streptomyces* and *Escherichia coli* strains used in this work are listed in Table 1. Spores were harvested from SFM solid plates [26] after growth at 30 °C for 7 days. In the bald strains, mycelium was collected from SFM cultures at 7 days. Differentiation analyses were carried out on GYM [27] (5 g/L glucose, 4 g/L yeast extract, 5 g/L malt extract, 0.5 g/L MgSO_4_.7H_2_O, 20 g/L agar, after autoclaving supplemented with sterile 0.5 g/L K_2_HPO_4_) plates covered with cellophane, inoculated with 10^7^ spores from a freshwater suspension and cultured at 30 °C. In the bald strains, an aliquot of the mycelium collected from 7-day-old SFM plates was used to quantify the protein per mL, and plates were inoculated with mycelium equivalent to 26.3 mg protein. Samples for quantification of actinorhodin and undecylprodigiosin production were obtained from 20 mL sucrose-free R5A [28] cultures grown at 30 °C and 200 rpm in 100 mL flasks inoculated with 10^7^ spores/mL, or with mycelium equivalent to 26.3 mg protein/mL in the bald strains. The bald phenotype was analysed in TBO [29], SFM and MM supplemented with 20% mannitol [26]. When indicated, plates were amended with 50 µg/mL methionine.

*Escherichia coli* strains were cultured in LB and 2xTY media at 37 °C. The following antibiotics were added to select plasmid-bearing and mutant strains: ampicillin (100 μg/mL), apramycin (100 μg/mL for *E. coli*, 25 µg/mL for *S. coelicolor*), hygromycin (100 μg/mL for *E. coli*, 200 µg/mL for *S. coelicolor*), kanamycin (50 μg/mL), thiostrepton (1 μg/mL) and nalidixic acid (25 µg/mL) (all from Sigma Aldrich Aldrich, Burlington, MA, USA).

### 4.2. SCO2103 Mutagenesis

The *SCO2103* knockout (Δ*SCO2103*) was created using the system developed by Tong et al. [34]. The 20 nt target sequence was selected inside *SCO2103* and amplified by PCR with the primers sg2103F and sgRNA-R (Table 1). The product, which was 1820 bp-long, was digested with *Nco*I/*SnaB*I and cloned in *Nco*I/*SnaB*I-digested pCRISPR-Cas9. The regions surrounding SCO2103 were amplified by PCR with 2103 leftF/2103 LeftR and 2103RightF/ 2103RightR primers. The DNA fragments were combined by overlap-extension PCR [36] with the primers 2103 leftF2103/2103RighR. The PCR product was cloned and sequenced in pCR^TM^-Blunt II-TOPO, followed by sequencing with primers M13F and M13R to confirm the absence of mutations. The insert was released with *Stu*I and cloned into pCRISPR-Cas9 harbouring the 20 nt target sequence digested with *Stu*I. The final vector pCRISPR-2103 was introduced into the *S. coelicolor* wild-type strain by conjugation. The conjugants harbouring the plasmid were selected using apramycin resistance, plated in SFM and grown at 37ºC for 3 days for plasmid clearance. Mutations were confirmed by PCR using the primers CAS91F and CAS91R (Table 1).

### 4.3. SCO2102 Mutagenesis

The *SCO2102* knockout (Δ*SCO2102* mutant) was created following the workflow described above for the Δ*SCO2102* mutant: the 20 nt target sequence was selected inside the *SCO2102* gene and amplified by PCR with the primers sg2102F and sgRNA-R. The regions surrounding SCO2102 were amplified by PCR with 2102 leftF/2102 LeftR and 2102 RightF/ 2102 RightR primers, and combined by overlap-extension PCR [36] with the primers 2102 leftF/ 2102 RightR. Fragments were cloned to create the pCRISPR-2102 plasmid, which was introduced into the *S. coelicolor* wild-type strain by conjugation; conjugants were selected, the plasmid was eliminated by growing the strain at 37 °C, and the mutations were confirmed by PCR using the primers CAS91F and CAS91R (Table 1).

### 4.4. Complementation of ΔSCO2102 and ΔSCO2103 Mutations

The integrative plasmid pNG3 [32] was used to introduce *SCO2102* and/or *SCO2103* with one or two promoters into the *SCO2102-03* mutants. The specific complementation constructions cloned in pNG3 were as follows: p_1_*SCO2103-02*, p_1_*SCO2103*, p_2_*SCO2102* p_1_p_2_*SCO2102* and p_1_-conditional terminator p_2_*SCO2102* (Figure 2f). 

p_1_*SCO2103-02*, p_1_*SCO2103* and p_2_*SCO2102* genes were amplified via PCR using Phusion High-Fidelity DNA Polymerase (Thermo). p_1_*SCO2103-02* was amplified with primers 2103F and 210203R, p_1_*SCO2103* with primers 2103F and 2103R, and p_2_*SCO2102* with primers SCO2102F and SCO2102R. Amplicons were cloned into pCR™-Blunt II-TOPO^®^, sequenced with primers M13F and M13R to confirm the absence of mutations, liberated with *EcoR*V and *Spe*I, and cloned into pNG3 [32] digested with *EcoR*V and *Spe*I. 

Synthesis of the p_1_p_2_*SCO2102* gene was ordered to BGI Genomics (Hong Kong). This synthetic gene had the *Spe*I recognition sequence, 294 bps including p1 (from *S. coelicolor* chromosomal position 2,262,210 to 2,261,917), 236 bps including p2 (from *S. coelicolor* chromosomal position 2,261,172 to 2,260,937), the SCO2102 ORF plus 63 bps (from *S. coelicolor* chromosomal position 2,260,936 to 2,259,881), and the *EcoR*V recognition sequence. The synthesised gene was cloned into pNG3 [32] between *EcoR*V and *Spe*I.

The p_1_-conditional terminator p_2_*SCO2102* was constructed as follows. A DNA fragment containing the conditional terminator and p2-*SCO2102* was amplified using primers TerF/R and cloned into pCR^TM^-Blunt II-TOPO. The conditional terminator was released from pCR^TM^-Blunt II-TOPO using *EcoR*V and *Xba*I and cloned into pKMV harbouring p1 digested with the same enzymes (pKMV-p1 was synthetised by BGI Genomics, Hong Kong) (Figure S3). The p_1_-conditional terminator p_2_*SCO2102* was cloned into pNG3 at the *Spe*I and *EcoR*V cloning sites.

The pNG3 plasmids harbouring the genes used for complementation were introduced into the Δ*SCO2103* bald mutant by protoplast transformation, as described in Kieser et al. [26]. Regarding the Δ*SCO2102* mutant, conjugation of the spores using *E. coli* ET12567/pUZ8002 as a donor strain was used to introduce the pNG3 plasmids following the protocol described by Kieser et al. [26]. The integration of these plasmids into the ɸBT1 integration site (gene *SCO4848*) [37] was verified by PCR using primers SCO4848F and pMS82R.

### 4.5. SCO2102/2103 and DnaA Sequence Analyses

The *S. coelicolor*, *S. griseus*, *S. avermitillis*, *S. lividans*, and *S. venezuelae SCO2102/2103* orthologue and the *S. coelicolor dnaA* (SCO3879) sequences were obtained from the StrepDB “http://strepdb.streptomyces.org.uk/” (accessed on 3 February 2022). Nucleotide similarities were estimated using the software package Lalign “http://www.ch.embnet.org/software/LALIGN_form.html” (accessed on 3 February 2022). Sequence alignments were performed using the MUSCLE software available from the free online platform Phylemon “http://phylemon.bioinfo.cipf.es/” (accessed on 15 January 2022). The homology plots shown in Figure S1 were created using Jalview 2.11.0 software. The putative, conditional, transcriptional/antiterminator conformations were predicted using the online algorithm developed by Millman et al. [18].

### 4.6. DNA and RNA Extraction

Genomic DNA isolation was performed following standard methods [26]. RNA was isolated using Direct-zol™ RNA columns (Zymo-Spin™, Irvine, CA, USA) and treated with DNase I (Qiagen, Germany). The quantity and integrity of the RNA samples were measured with Nanodrop 2000 (Thermo Scientific, Waltham, MA, USA) and 2100 Bioanalyzer (Agilent, Santa Clara, CA, USA).

### 4.7. Real-Time Quantitative Reverse-Transcription PCR (qRT-PCR)

A High-Capacity cDNA Reverse-Transcription Kit (Applied Biosystems, Waltham, MA, USA) was used to synthetise cDNA from 0.5 μg of RNA from three biological replicates. Real-time PCRs were carried out on a fluorescent quantitative-detection system FQD-96A (BIOER). The reactions were performed in triplicate, using 2 μL of two-fold diluted cDNA, 10 μL of PowerTrack™ SYBR Green Master Mix (ThermoFisher, Bremen, Germany), and 300 nM of specific primers (listed in Table 1) in a final volume of 20 μL. *SCO2102* and *SCO2103* were amplified with q2102F/R and q2103F/R primers, respectively; SCO4758 (amplified using primers q4758F/R) was used as a control since its expression is constitutive during development [20]. DNA contamination and primer dimer amplification were tested in negative controls by replacing cDNA with RNA or water. The amplification conditions were as follows: 2 min at 50 °C, 10 min at 95 °C, 40 repetitions of 15 s at 95 °C and 1 min at 60 °C.

Primer efficiencies were measured using serial dilutions of genomic DNA as a template and differed between the two primer pairs used. Consequently, we performed absolute quantification generating standard quantification curves using PCR-amplified *SCO2102/03* and *SCO4758* DNA fragments as templates for each primer pair [38]. All the standard curves used had a linear correlation coefficient greater than 0.99. Three biological replicates were processed. Statistical significance was determined by comparing transcript abundances using a *t*-test.

### 4.8. Antibiotic Quantification

Undecylprodigiosin and actinorhodin were quantified spectrophotometrically, according to Tsao et al. [39] and Bystrykh et al. [40]. For actinorhodin quantification, KOH was added to the culture samples at a final concentration of 1N. Cellular pellets were discarded by centrifugation, and the actinorhodin concentration was spectrophotometrically determined at 640 nm, applying the linear Beer–Lambert relationship (*ε*_640_ = 25,320). The culture samples for undecylprodigiosin quantification were lyophilised, resuspended in methanol, acidified with 0.5N HCl and spectrophotometrically assayed at 530 nm, using the Beer–Lambert relationship to estimate the concentrations (*ε*_530_ = 100,500). Three biological replicates were processed. Antibiotic concentrations were normalised by total protein. Three biological replicates were analysed. The reliability of the differences in antibiotic production compared to the wild-type strain was analysed using Student’s *t*-tests. Differences were considered significant if the *p* value was equal to or less than 0.05 (asterisks in Figure 1d and Figure 4m–r).

### 4.9. Protein Quantification

Protein concentration was quantified by the Bradford [41] method using bovine serum albumin standard (Sigma Aldrich, Burlington, MA, USA). Total protein extracts were obtained by mixing a volume of culture with a volume of 1 M NaOH, boiling for 5 min and removing cell debris by centrifugation at 7740× *g*.

### 4.10. S. coelicolor SCO2102 mCherry/eGFP and SCO2103 eGFP Constructions

We designed the SCO2102 ORF including the P2 promoter (Figure 1a) together with the SCO2102 and the mCherry ORFs (Figure S3). In addition, we designed an eGFP ORF (Figure S3). The *eGFP* and *mCherry* codon usages were manually optimised to *Streptomyces*, using the *Streptomyces* codon usage table from 100 *Streptomyces* genes reported by Kieser et al. [26]. These genes were synthesised by BGI Genomics (Hong Kong), who cloned them into pMV. *P2-SCO2102-mCherry* was released from pMV using *Spe*I and *EcoR*V and cloned into pNG3 [32] digested with the same enzymes. The pNG3 harbouring *P2-SCO2102-eGFP* was created by releasing *mCherry* using *Xho*I and *EcoR*V and replacing it with eGFP released from pMV with the same enzymes.

*SCO2103-eGFP* was created as follows. P1-*SCO2103* was amplified using primers 2103F/2103R2 and cloned into pCRTM-Blunt II-TOPO; P1-SCO2103 was released using *Spe*I and *Xho*I and cloned into pNG3 harbouring p2-*SCO2102*-eGFP digested with the same enzymes. Eventually, P1-SCO2103-eGFP was cloned into pRAS (plasmid derived from pRA loaned by Antonio Rodríguez and Alberto Sola-Landa; INBIOTEC, León, Spain) [33] at the *Spe*I and *EcoR*V cloning sites. The integration of pRAS into the ɸC31 integration site (gene *SCO3798*) [42] was verified by PCR using primers SCO3798intF and SCO3798intR.

The above pRAS- and pNG3-integrative plasmids were introduced to the *S. coelicolor* wild-type or *ΔSCO2102* strains via conjugation [26].

### 4.11. Viability Staining, mCherry and eGFP Visualisation

Culture samples were obtained and processed to analyse cell viability and sporulation at various incubation time points, as previously described [43]. Cell viability and sporulation were analysed by staining the cells with propidium iodide and SYTO 9 (LIVE/DEAD Bac-Light Bacterial Viability Kit, Invitrogen, L-13152, Waltham, MA, USA). The samples were observed under a Leica TCS-SP8 confocal laser-scanning microscope with wavelengths of 488 and 568 nm excitations and 530 nm (green) or 640 nm (red) emissions [43] for SYTO 9/PI staining.

SCO2102-mCherry and SCO2103-eGFP were analysed in SFM plates without cellophane. After spore inoculation, cover glasses were positioned at an angle of 45 degrees. At the indicated time-points, the cover glasses were removed and the samples were mounted with ultrapure mQ water. Then, they were observed using a Leica DMRXA fluorescence microscope with FITC and Texas Red filters. Pictures were taken with an ORCA-Flash4.0 V3 Digital CMOS camera. 

Culture controls without SYTO 9/PI staining and without eGFP or mCherry were used to fix the levels at which autofluorescence was detected.

Microscopy images were processed (histogram intensity levels were adjusted, and the scale was added) using Fiji software [44]. Figure composites were made using AdobePhotoshop CS5.1.

### 4.12. Quantification of Chromosomal DNA

Hyphae were resuspended in Tris-HCl 20 mM, pH 8, EDTA 1mM, complete mini EDTA-free (Roche) protease inhibitor cocktail, and broken via sonication (MSE soniprep 150) in an ice-bath for 6 cycles comprising 10 s of power and a 60-second break. Cellular debris was removed by centrifugation (10,000g for 10 min). Chromosomal DNA was quantified using the Burton assay, hydrolysing DNA with perchloric acid and quantifying deoxyribose [45,46]. The DNA standard curves were created using a 1 mg/mL calf thymus DNA (Sigma Aldrich) stock solution. The DNA concentration was normalised relative to the total protein measured by Bradford [41], and samples were analysed in triplicate; statistical significance was estimated using a *t*-test.

## Figures and Tables

**Table 1:** Bacterial strains, plasmids and primers used in this study.

Strain	Description	Reference
*S.coelicolor* M145	SCP1^-^ SCP2^-^, reference strain	[26]
*S. coelicolor* Δ*SCO2102*	*SCO2102* eliminated by CRISPR−Cas9	This study
*S. coelicolor* Δ*SCO2103*	*SCO2102* eliminated by CRISPR−Cas9	This study
*E. coli* TOP10	F^-^ *mcr*A Δ (*mrr*−*hsd*RMS−*mcr*BC) φ80*lac*ZΔM15 Δ*lac*X74 *rec*A1 *ara*D139 Δ (*ara*-*leu*)7697 *gal*U *gal*K *rps*L *end*A1 *nup*G	Invitrogen
*E. coli* ET12567	*dam-13::*Tn9*, dcm-6, hsdM*, *hsdR*	[30]
*E. coli* ET12567/pUZ8002	*E. coli* ET12567 harbouring pUZ8002, a non-self-transmissible plasmid which can mobilize *oriT*−containing plasmids by conjugation	[31]
**Plasmids**	**Description**	**Reference**
pMV	*Cloning vector; Amp^R^*	BGI
pKMV	*Cloning vector; Km^R^*	BGI
pNG3	Integrative and conjugative vector, Hygro^R^	[32]
pRAS	pRA modified by Antonio Rodríguez and Alberto−Sola−Landa	[33]
pNG3 p2−*SCO2102*	pNG3 harbouring p2−*SCO2102*, Hygro^R^	This study
pNG3 p1−p2−*SCO2102*	pNG3 harbouring p1−p2−*SCO2102*, Hygro^R^	This study
pNG3 *SCO2103*	pNG3 harbouring *SCO2103*, Hygro^R^	This study
pNG3 *SCO2102-03*	pNG3 harbouring *SCO2102* and *SCO2103*, Hygro^R^	This study
PCR™-Blunt II−TOPO^®^	Zero Blunt^®^ TOPO^®^ PCR Cloning Kit, Kan^R^	Invitrogen
pCRISPR−Cas9	Conjugative and thermosensitive plasmid harbouring Cas9	[34]
pCRISPR−2102	pCRISPR harbouring the target *SCO2102* sequence and the 2.13 kb fragment used to create the *SCO2102* knockout	This study
pCRISPR−2103	pCRISPR harbouring the target *SCO2103* sequence and the 2.61 kb fragment used to create the *SCO2103* knockout	This study
pNG3−p2−SCO2102−mCherry	pNG3 harbouring *SCO2102,* mCherry and p2	This study
pNG3−p2−SCO2102-eGFP	pNG3 harbouring *SCO2102,* eGFP and p2	This study
pRAS−p1−SCO2103−eGFP	pRAS harbouring p1, *SCO2103* and eGFP	This study
pNG3−p1−conditional terminator−p2−SCO2102	pNG3 harbouring p1, the conditional terminator, p2 and *SCO2102*	This study
**Primer**	**Sequence**	**Reference**
SCO2102F	AAAAAGATATCCTCACAGCCAAGGACGATCC	This study
SCO2102R	CCCACTAGTGGACTCGTGGTGGAGGGG	This study
SCO4848F	CGTCGTATCCCCTCGGTTG	[35]
pMS82R	GAGCCGGGAAAGCTCATTCA	[35]
M13 F	CAGGAAACAGCTATGA	Invitrogen
M13 R	CTGGCCGTCGTTTTAC	Invitrogen
2102 leftF	CGGTTGGGTGACCGCCGCA	This study
2102leftR	GCGGCTCCGGCTTCCTCTTCCGTACGCCCCTCTCCCAGTGGC	This study
2102 rightF	GCCACTGGGAGAGGGGCGTACGGAAGAGGAAGCCGGAGCCGC	This study
2102 rightR	AGGAGCCGCTGTGGCCCAAC	This study
sg2102F	GGCTTCCTCTTCCTCAGAAG	This study
sgRNA−R	ACGCCTACGTAAAAAAAGCACCGACTCGGTGCC	This study
CAS91R	GTAGTACGGGATGCGGAAGG	This study
CAS9−1F	ATTACTGGACCGGATCGGG	This study
2103F	AACCTGGACGAGGTGCTGG	This study
2103R	CGTTACGCAGTGTGTCGCAAAT	This study
210203R	TGCGCGACCGTCTCCTGT	This study
sg2103F	CCGGCGGCTGTACGCTCGAT	This study
2103 LeftF	GTAGCCAGGTTTCCGACTGT	This study
2103 LeftR	GATCGTCCTTGGCTGTGAGATGGGCAGGTTAGCCAGGGT	This study
2103 RightF	ACCCTGGCTAACCTGCCCAT CTCACAGCCAAGGACGATC	This study
2103 RightR	TCTTCCTCAGAAGCGGTACT	This study
TerF	AACTGGTCAGGCTCATCAAGG	This study
TerR	TTCCGCGGCTCCCAGT	This study
2103R2	GGCCCGCGGTGGGTGG	This study
q2103F	GGTGACCAGTGTGAAGATG	This study
q2103R	GAGTTGTTGAGCGTGATGA	This study
q2102F	AACTCGTACGGCGTCTA	This study
q2102R	ATGTACGGGTCGGAGTAG	This study
q4758F	ATCACCGACCGGATGCCCTT	[20]
q4758R	GCCGAGCCCCGCTTCTTC	[20]
SCO3798intF	CAGCTCGTCCTTGGTGTTCA	This study
SCO3798intR	TCAGGTCCATGACGTTTCCC	This study

## Data Availability

Not applicable.

## Supplementary Materials

The following supporting information can be downloaded at: https://www.mdpi.com/article/10.3390/ijms23094984/s1.

## References

[1] Berdy J. (2005). Bioactive microbial metabolites. J. Antibiot. (Tokyo).

[2] Hopwood D.A. (2007). Streptomyces in Nature and Medicine: The Antibiotic Makers.

[3] Flardh K., Buttner M.J. (2009). Streptomyces morphogenetics: Dissecting differentiation in a filamentous bacterium. Nat. Rev. Microbiol..

[4] Yague P., Lopez-Garcia M.T., Rioseras B., Sanchez J., Manteca A. (2013). Pre-sporulation stages of Streptomyces differentiation: State-of-the-art and future perspectives. FEMS Microbiol. Lett..

[5] Jakimowicz D., van Wezel G.P. (2012). Cell division and DNA segregation in Streptomyces: How to build a septum in the middle of nowhere?. Mol. Microbiol..

[6] Saxena R., Fingland N., Patil D., Sharma A.K., Crooke E. (2013). Crosstalk between DnaA protein, the initiator of escherichia coli chromosomal replication, and acidic phospholipids present in bacterial membranes. Int. J. Mol. Sci..

[7] Zawilak-Pawlik A., Nowaczyk M., Zakrzewska-Czerwińska J. (2017). The role of the N-terminal domains of bacterial initiator DnaA in the assembly and regulation of the bacterial replication initiation complex. Genes.

[8] Lebkowski T., Wolanski M., Oldziej S., Flardh K., Zakrzewska-Czerwinska J. (2020). AfsK-mediated site-specific phosphorylation regulates DnaA initiator protein activity in streptomyces coelicolor. J. Bacteriol..

[9] Blaauwen T.d., Hamoen L.W., Levin P.A. (2017). The divisome at 25: The road ahead. Curr. Opin. Microbiol..

[10] Plachetka M., Zyla-Uklejewicz D., Weigel C., Donczew R., Donczew M., Jakimowicz D., Zawilak-Pawlik A., Zakrzewska-Czerwinska J. (2019). Streptomycete origin of chromosomal replication with two putative unwinding elements. Microbiology.

[11] McCormick J.R., Su E.P., Driks A., Losick R. (1994). Growth and viability of Streptomyces coelicolor mutant for the cell division gene ftsZ. Mol. Microbiol..

[12] McCormick J.R. (2009). Cell division is dispensable but not irrelevant in Streptomyces. Curr. Opin. Microbiol..

[13] Willemse J., Borst J.W., de Waal E., Bisseling T., van Wezel G.P. (2011). Positive control of cell division: FtsZ is recruited by SsgB during sporulation of Streptomyces. Genes Dev..

[14] Del Sol R., Mullins J.G., Grantcharova N., Flardh K., Dyson P. (2006). Influence of CrgA on assembly of the cell division protein FtsZ during development of Streptomyces coelicolor. J. Bacteriol..

[15] Cantlay S., Sen B.C., Flärdh K., McCormick J.R. (2021). Influence of core divisome proteins on cell division in Streptomyces venezuelae ATCC 10712. Microbiology.

[16] Blanco J., Coque J.J., Martin J.F. (1998). The folate branch of the methionine biosynthesis pathway in Streptomyces lividans: Disruption of the 5,10-methylenetetrahydrofolate reductase gene leads to methionine auxotrophy. J. Bacteriol..

[17] Shetty S., Varshney U. (2020). Regulation of translation by one-carbon metabolism in bacteria and eukaryotic organelles. J. Biol. Chem..

[18] Millman A., Dar D., Shamir M., Sorek R. (2017). Computational prediction of regulatory, premature transcription termination in bacteria. Nucleic Acids Res..

[19] Jeong Y., Kim J.-N., Kim M.W., Bucca G., Cho S., Yoon Y.J., Kim B.-G., Roe J.-H., Kim S.C., Smith C.P. (2016). The dynamic transcriptional and translational landscape of the model antibiotic producer Streptomyces coelicolor A3(2). Nat. Commun..

[20] Li S., Wang W., Li X., Fan K., Yang K. (2015). Genome-wide identification and characterization of reference genes with different transcript abundances for Streptomyces coelicolor. Sci. Rep..

[21] Willemse J., van Wezel G.P. (2009). Imaging of Streptomyces coelicolor A3 with reduced autofluorescence reveals a novel stage of FtsZ localization. PLoS ONE.

[22] Yagüe P., Willemse J., Koning R.I., Rioseras B., López-García M.T., Gonzalez-Quiñonez N., Lopez-Iglesias C., Shliaha P.V., Rogowska-Wrzesinska A., Koster A.J. (2016). Subcompartmentalization by cross-membranes during early growth of Streptomyces hyphae. Nat. Commun..

[23] Kim D., Huh J.-H., Yang Y.-Y., Kang C.-M., Lee I.-H., Hyun C.-G., Hong S.-K., Suh J.-W. (2003). Accumulation of S -Adenosyl -l- Methionine Enhances Production of Actinorhodin but Inhibits Sporulation in Streptomyces lividans TK23. J. Bacteriol..

[24] Manteca A., Yague P. (2018). Streptomyces Differentiation in Liquid Cultures as a Trigger of Secondary Metabolism. Antibiotics.

[25] Plachetka M., Krawiec M., Zakrzewska-Czerwinska J., Wolanski M. (2021). AdpA positively regulates morphological differentiation and chloramphenicol biosynthesis in Streptomyces venezuelae. Microbiol. Spectr..

[26] Kieser T. (2000). Practical Streptomyces Genetics.

[27] Novella I.S., Barbés C., Sánchez J. (1992). Sporulation of Streptomyces antibioticus ETHZ 7451 in submerged culture. Can. J. Microbiol..

[28] Sanchez C., Butovich I.A., Brana A.F., Rohr J., Mendez C., Salas J.A. (2002). The biosynthetic gene cluster for the antitumor rebeccamycin: Characterization and generation of indolocarbazole derivatives. Chem. Biol..

[29] Aparicio J.F., Colina A.J., Ceballos E., Martin J.F. (1999). The biosynthetic gene cluster for the 26-membered ring polyene macrolide pimaricin. A new polyketide synthase organization encoded by two subclusters separated by functionalization genes. J. Biol. Chem..

[30] MacNeil D.J., Gewain K.M., Ruby C.L., Dezeny G., Gibbons P.H., MacNeil T. (1992). Analysis of Streptomyces avermitilis genes required for avermectin biosynthesis utilizing a novel integration vector. Gene.

[31] Flett F., Mersinias V., Smith C.P. (1997). High efficiency intergeneric conjugal transfer of plasmid DNA from Escherichia coli to methyl DNA-restricting streptomycetes. FEMS Microbiol. Lett..

[32] Gonzalez-Quinonez N., Lopez-Garcia M.T., Yague P., Rioseras B., Pisciotta A., Alduina R., Manteca A. (2016). New PhiBT1 site-specific integrative vectors with neutral phenotype in Streptomyces. Appl. Microbiol. Biotechnol..

[33] Perez-Redondo R., Santamarta I., Bovenberg R., Martin J.F., Liras P. (2010). The enigmatic lack of glucose utilization in Streptomyces clavuligerus is due to inefficient expression of the glucose permease gene. Microbiology.

[34] Tong Y., Charusanti P., Zhang L., Weber T., Lee S.Y. (2015). CRISPR-Cas9 Based Engineering of Actinomycetal Genomes. ACS Synth. Biol..

[35] Rioseras B., Yagüe P., García M.T.L., Gonzalez-Quiñonez N., Binda E., Marinelli F., Manteca A. (2016). Characterization of SCO4439, a D-alanyl-D-alanine carboxypeptidase involved in spore cell wall maturation, resistance and germination in Streptomyces coelicolor. Sci. Rep..

[36] Lee J., Shin M.K., Ryu D.K., Kim S., Ryu W.-S., Braman J. (2010). Insertion and deletion mutagenesis by overlap extension PCR. sertion and deletion mutagenesis by overlap extension PCR. In In Vitro Mutagenesis Protocols.

[37] Gregory M.A., Till R., Smith M.C. (2003). Integration site for Streptomyces phage phiBT1 and development of site-specific integrating vectors. J. Bacteriol..

[38] Rutledge R.G., Cote C. (2003). Mathematics of quantitative kinetic PCR and the application of standard curves. Nucleic Acids Res..

[39] Tsao S.W., Rudd B.A., He X.G., Chang C.J., Floss H.G. (1985). Identification of a red pigment from Streptomyces coelicolor A3 as a mixture of prodigiosin derivatives. J. Antibiot. (Tokyo).

[40] Bystrykh L.V., Fernandez-Moreno M.A., Herrema J.K., Malpartida F., Hopwood D.A., Dijkhuizen L. (1996). Production of actinorhodin-related "blue pigments" by Streptomyces coelicolor A3. J. Bacteriol..

[41] Bradford M.M. (1976). A rapid and sensitive method for the quantitation of microgram quantities of protein utilizing the principle of protein-dye binding. Anal. Biochem..

[42] Combes P., Till R., Bee S., Smith M.C. (2002). The streptomyces genome contains multiple pseudo-attB sites for the (phi)C31-encoded site-specific recombination system. J. Bacteriol..

[43] Manteca A., Alvarez R., Salazar N., Yague P., Sanchez J. (2008). Mycelium differentiation and antibiotic production in submerged cultures of Streptomyces coelicolor. Appl. Environ. Microbiol..

[44] Schindelin J., Arganda-Carreras I., Frise E., Kaynig V., Longair M., Pietzsch T., Preibisch S., Rueden C., Saalfeld S., Schmid B. (2012). Fiji: An open-source platform for biological-image analysis. Nat. Methods.

[45] Burton K. (1956). A study of the conditions and mechanism of the diphenylamine reaction for the colorimetric estimation of deoxyribonucleic acid. Biochem. J..

[46] Waterborg J.H., Matthews H.R. (1985). The burton assay for DNA. Methods Mol. Biol..

