# Supplementary material for: The SCO2102 Protein Harbouring a DnaA II Protein-Interaction Domain Is Essential for the SCO2103 Methylenetetrahydrofolate Reductase Positioning at Streptomyces Sporulating Hyphae, Enhancing DNA Replication during Sporulation
Source: Int J Mol Sci. 2022 Apr 30;23(9):4984. doi: 10.3390/ijms23094984 (PMC9099993; doi:10.3390/ijms23094984)

Figure S1. *S. coelicolor* DnaA domain II and SCO2102 DnaA domain conservation. The homology plots were created using the Jalview 2.11.0 software.

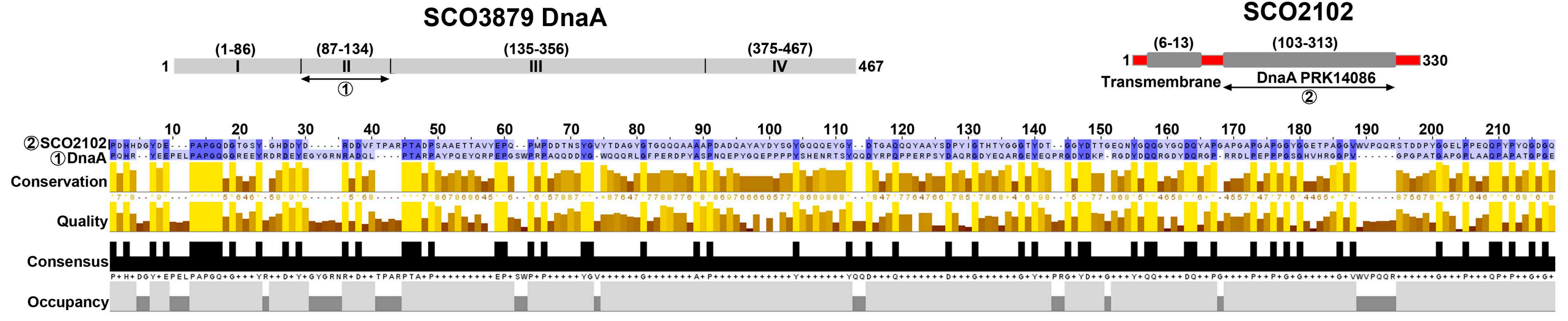

Supplement: Supplementary file 1 [file ijms-23-04984-s001.zip › Figure S1.pdf]
