# Supplementary material for: The SCO2102 Protein Harbouring a DnaA II Protein-Interaction Domain Is Essential for the SCO2103 Methylenetetrahydrofolate Reductase Positioning at Streptomyces Sporulating Hyphae, Enhancing DNA Replication during Sporulation
Source: Int J Mol Sci. 2022 Apr 30;23(9):4984. doi: 10.3390/ijms23094984 (PMC9099993; doi:10.3390/ijms23094984)

Figure S2. Control cultures of the *S. coelicolor* wild-type strain showing the absence of autofluorescence under the culture conditions, developmental time-points and the microscopy settings used to follow the FtsZ-eGFP, SCO2102-mCherry and SCO2103-eGFP kinetic shown in Figures 7 and 8. Scale bars indicate 5 μm.

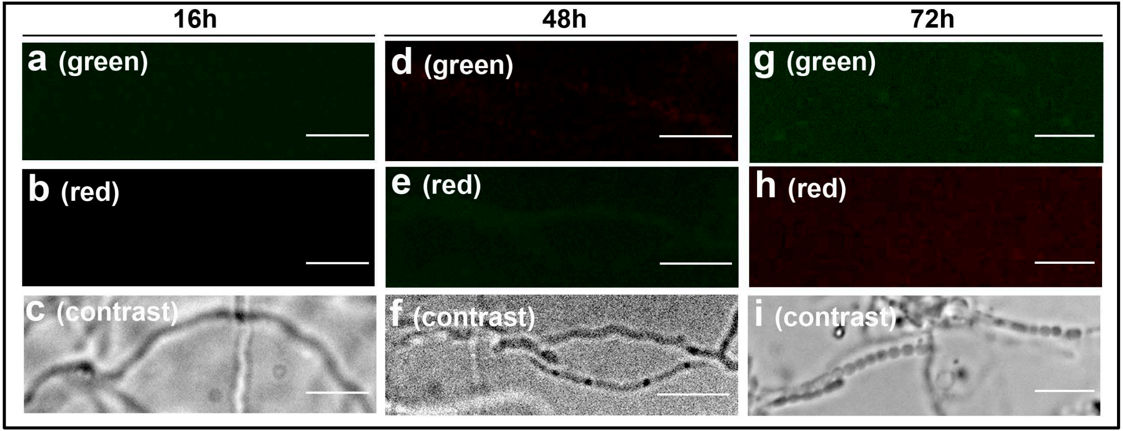

Supplement: Supplementary file 1 [file ijms-23-04984-s001.zip › Figure S2.pdf]
