# Supplementary material for: The SCO2102 Protein Harbouring a DnaA II Protein-Interaction Domain Is Essential for the SCO2103 Methylenetetrahydrofolate Reductase Positioning at Streptomyces Sporulating Hyphae, Enhancing DNA Replication during Sporulation
Source: Int J Mol Sci. 2022 Apr 30;23(9):4984. doi: 10.3390/ijms23094984 (PMC9099993; doi:10.3390/ijms23094984)
Supplement: Supplementary file 1 [file ijms-23-04984-s001.zip › Figure S3.pdf]

Figure S3. DNA sequences synthetised. The *eGFP* and *mCherry* codon usages were manually optimised to *Streptomyces*, using the Streptomyces codon usage table of 100 *Streptomyces* genes reported by Kieser et al. [1].

P1 281 bps

*Spe*I + P1 + *Xba*I + *Eco*RV

ACTAGT
AACCTGGACGAGGTGCTGGAGGCGGGCGCCCGGCGCGTGGTCGTCGTGCGCGCGA
TCACCGCCGCCGAGGACCCGGGCGCGGCGGCGGCCGAGTTCGCCCGGCGGCTGCG
GCAGGCTCCGGCACACGGGTAGCCGCACCTCGCCACCGACGTCGTCGCAGGCCGTC
GTCTGCGGCGGGCAGGTGTCCAAAGGGTGGACAACAACTCGACAATGTGGACAAA
AGTCCCGCATCCGGTTGGGTGACCGCCGCACCCTGGCTAACCTGCCCAT TCTAGA
GATATC

P1-P2-SCO2102 1574 bps

*Spe*I + P1 + P2 + *SCO2102* + *Eco*RV

ACTAG
TAACCTGGACGAGGTGCTGGAGGCGGGCGCCCGGCGCGTGGTCGTCGTGCGCGCG
ATCACCGCCGCCGAGGACCCGGGCGCGGCGGCGGCCGAGTTCGCCCGGCGGCTGC
GGCAGGCTCCGGCACACGGGTAGCCGCACCTCGCCACCGACGTCGTCGCAGGCCGT
CGTCTGCGGCGGGCAGGTGTCCAAAGGGTGGACAACAACTCGACAATGTGGACAA
AAGTCCCGCATCCGGTTGGGTGACCGCCGCACCCTGGCTAACCTGCCCATCTCACA
GCCAAGGACGATCCGGCGGCTGTACGCTCGATCGGCATCGAGTTCGCCACGGAGTT
CTGCGCGCGGCTGCTGGCCGAGGGAGTGCCAGGACTGCACTTCATCACGCTCAACA
ACTCCACGGCGACGCTGGAAATCTACGAGAACCTGGGCCTGCACCACCCACCGCGG
GCCTAGACCGGCCGCACGTATTTGCGACACACTGCGTAACGGCCACTGGGAGAGGG
GCGTACATGGGCTGGACGGTCCTCTACATCGCGTTCGGCGTCGTCGCGCTGTGGCT
GCTCGGCGAGGTGCTGCTGCAGTACAAGGCGCGGCTGCGCTGGCGGCTGCTGGCCT
TCGCCGGCTTCGTCGGCGTCGTGGCCGGTGTGCTGATGTCGAACGTGCTCGTCATCG
GCGTCGGTGCCGCCGCCTTCGCGGTCGGCCAGACCTACGTCACCCTGTCGTTCCGCC
GCGGCTTCGAGGCCGGCTGGGCGGTCAACGCCCCGGCGAGCCTCGTCGGCAAGCGC
GGGCGTCCCGAACGGGGCCGCCGGGAACCGACGTTGGAGGTCTCCGGGCTCGAAC
CCGCCGAGGGCGGCCCGGACCACCACGACGGCTACGACGAGCCCGCCCCGGGCCA
GGACGGCACCGGCTCGTACGGGCACGACGACTACGACCGCGACGACGTCTTCACCC
CGGCCCGGCCCACCGCCGACCCCTCGGCCGCAGAGACCACCGCCGTCTACGAACCG
CAGCCCATGCCGGACGACACCAACTCGTACGGCGTCTACACCGACGCCGGTTACGG
CACCGGCCAGCAGCAGGCGGCCGCCGCGCCGGACGCCGACCAGGCGTACGCCTAC
GACTACTCCGGCTACGGCCAGCAGCAGGAGTACGGCTACGACACCGGCGCCCAGC
AGCAGTACGCCGCCTACTCCGACCCGTACATCGGCACCCACACCTACGGCGGCGGG
ACGTACGACACCGGCGGCTACGACACGACCGGTGAGCAGAACTACGGCCAGCAGG
GCTACGGGCAGGACCAGTACGCCCCCGGCGCCCCCGGCGCCCCCGGCGCGCCCGGC
GGCTACGGCGGCGAGACCCCGGCCGGCGGAGTGTGGGTGCCGCAGCAGCGCAGCA

CCGACGACCCCTACGGCGGCGAGCTCCCGCCCGAGCAGCAGCCCTACCCCTACCAG
GGCGACGGCCAGACGCAGGGCCAGGGCTACGACGAGCAGTACCGCTTCTGAGGAA
GAGGAAGCCGGAGCCGCTCACTGGGAGCCGCGGAACTCCGGCCCCTCCACCACGA
GTCC GATATC

P2-SCO2102-mcherry   1973 bps

*Spe*I + P2 + *Nde*I SCO2102 + *Xho*I + mcherry + *Eco*RV

ACTAGTATCCTCACAGCCAAGGACGATCCGGCGGCTGTACGCTCGATCGGCATCGA
GTTCGCCACGGAGTTCTGCGCGCGGCTGCTGGCCGAGGGAGTGCCAGGACTGCACT
TCATCACGCTCAACAACTCCACGGCGACGCTGGAAATCTACGAGAACCTGGGCCTG
CACCACCCACCGCGGGCCTAGACCGGCCGCACGTATTTGCGACACACTGCGTAACG
GCCACTGGGAGAGGGGCGTACCATATGGGCTGGACGGTCCTCTACATCGCGTTCGG
CGTCGTCGCGCTGTGGCTGCTCGGCGAGGTGCTGCTGCAGTACAAGGCGCGGCTGC
GCTGGCGGCTGCTGGCCTTCGCCGGCTTCGTCGGCGTCGTGGCCGGTGTGCTGATGT
CGAACGTGCTCGTCATCGGCGTCGGTGCCGCCGCCTTCGCGGTCGGCCAGACCTAC
GTCACCCTGTCGTTCCGCCGCGGCTTCGAGGCCGGCTGGGCGGTCAACGCCCCGGC
GAGCCTCGTCGGCAAGCGCGGGCGTCCCGAACGGGGCCGCCGGGAACCGACGTTG
GAGGTCTCCGGGCTCGAACCCGCCGAGGGCGGCCCGGACCACCACGACGGCTACG
ACGAGCCCGCCCCGGGCCAGGACGGCACCGGCTCGTACGGGCACGACGACTACGA
CCGCGACGACGTCTTCACCCCGGCCCGGCCCACCGCCGACCCCTCGGCCGCAGAGA
CCACCGCCGTCTACGAACCGCAGCCCATGCCGGACGACACCAACTCGTACGGCGTC
TACACCGACGCCGGTTACGGCACCGGCCAGCAGCAGGCGGCCGCCGCGCCGGACG
CCGACCAGGCGTACGCCTACGACTACTCCGGCTACGGCCAGCAGCAGGAGTACGGC
TACGACACCGGCGCCCAGCAGCAGTACGCCGCCTACTCCGACCCGTACATCGGCAC
CCACACCTACGGCGGCGGGACGTACGACACCGGCGGCTACGACACGACCGGTGAG
CAGAACTACGGCCAGCAGGGCTACGGGCAGGACCAGTACGCCCCCGGCGCCCCCG
GCGCCCCCGGCGCGCCCGGCGGCTACGGCGGCGAGACCCCGGCCGGCGGAGTGTG
GGTGCCGCAGCAGCGCAGCACCGACGACCCCTACGGCGGCGAGCTCCCGCCCGAG
CAGCAGCCCTACCCCTACCAGGGCGACGGCCAGACGCAGGGCCAGGGCTACGACG
AGCAGTACCGCTTCCTCGAGATGGTCAGCAAGGGCGAGGAGGACAACATGGCCAT
CATCAAGGAGTTCATGCGCTTCAAGGTCCACATGGAGGGCTCCGTCAACGGCCACG
AGTTCGAGATCGAGGGCGAGGGCGAGGGCCGCCCGTACGAGGGCACCCAGACCGC
CAAGCTGAAGGTCACCAAGGGCGGCCCGCTGCCGTTCGCCTGGGACATCCTGTCCC
CGCAGTTCATGTACGGCTCCAAGGCCTACGTCAAGCACCCCGCCGACATCCCGGAC
TACCTGAAGCTGTCCTTCCCCGAGGGCTTCAAGTGGGAGCGCGTCATGAACTTCGA
GGACGGCGGCGTCGTCACCGTCACCCAGGACTCCTCCCTGCAGGACGGCGAGTTCA
TCTACAAGGTCAAGCTCCGCGGCACCAACTTCCCGTCCGACGGCCCGGTCATGCAG
AAGAAGACCATGGGCTGGGAGGCCTCCTCCGAGCGCATGTACCCGGAGGACGGCG
CCCTGAAGGGCGAGATCAAGCAGCGCCTGAAGCTGAAGGACGGCGGCCACTACGA
CGCCGAGGTCAAGACCACCTACAAGGCCAAGAAGCCGGTCCAGCTGCCGGGCGCC
TACAACGTCAACATCAAGCTGGACATCACCTCCCACAACGAGGACTACACCATCGT
CGAGCAGTACGAGCGCGCCGAGGGCCGCCACTCCACCGGCGGCATGGACGAGCTG
TACAAGTGATACGTAGTTAACGATATC

---

eGFP 744 BPS

*Xho*I + eGFP + *Eco*RV

CTCGAGATGGTCTCCAAGGGCGAGGAGCTGTTCACCGGCGTCGTCCCGATCCTGGT
CGAGCTGGACGGCGACGTCAACGGCCACAAGTTCTCCGTCTCCGGCGAGGGCGAG
GGCGACGCCACCTACGGCAAGCTGACCCTGAAGTTCATCTGCACCACCGGCAAGCT
GCCGGTCCCGTGGCCGACCCTGGTCACCACCCTGACCTACGGCGTCCAGTGCTTCTC
CCGCTACCCGGACCACATGAAGCAGCACGACTTCTTCAAGTCCGCCATGCCGGAGG
GCTACGTCCAGGAGCGCACCATCTTCTTCAAGGACGACGGCAACTACAAGACCCGC
GCCGAGGTCAAGTTCGAGGGCGACACCCTGGTCAACCGCATCGAGCTGAAGGGCA
TCGACTTCAAGGAGGACGGCAACATCCTGGGCCACAAGCTGGAGTACAACTACAA
CTCCCACAACGTCTACATCATGGCCGACAAGCAGAAGAACGGCATCAAGGTCAACT
TCAAGATCCGCCACAACATCGAGGACGGCTCCGTCCAGCTGGCCGACCACTACCAG
CAGAACACCCCGATCGGCGACGGCCCGGTCCTGCTGCCGGACAACCACTACCTGTC
CACCCAGTCCGCCCTGTCCAAGGACCCCAACGAGAAGCGCGACCACATGGTCCTGC
TGGAGTTCGTCACCGCCGCCGGCATCACCCTGGGCATGGACGAGCTGTACAAGTGA
TACGTAGTTAACGATATC

---

1. Kieser, T., *Practical streptomyces genetics*. John Innes Foundation: Norwich, 2000.
